# Childbirth Experiences in the United Kingdom Compared to the Netherlands: A Cross‐Sectional Survey Study

**DOI:** 10.1111/birt.70006

**Published:** 2025-08-13

**Authors:** Lauri M. M. van den Berg, Jens Henrichs, Jeroen van Dillen, Soo Downe, Corine Verhoeven, Ank de Jonge

**Affiliations:** ^1^ Department of Midwifery Science Amsterdam University Medical Centre Amsterdam the Netherlands; ^2^ Midwifery Academy Amsterdam Groningen, Inholland Amsterdam the Netherlands; ^3^ Amsterdam Public Health Research Institute Amsterdam University Medical Centre Amsterdam the Netherlands; ^4^ Department of Obstetrics and Gynaecology Radboud University Medical Centre Nijmegen the Netherlands; ^5^ Faculty of Health and Care, School of Nursing and Midwifery University of Central Lancashire Preston UK; ^6^ Division of Midwifery, School of Health Sciences University of Nottingham Nottingham UK; ^7^ Amsterdam Reproduction and Development Research Institute Amsterdam the Netherlands

**Keywords:** care‐related factors, childbirth experience, Netherlands, respectful maternity care, socio‐demographic factors, survey, United Kingdom, woman‐centered care

## Abstract

**Introduction:**

This study was performed to compare childbirth experiences in the United Kingdom (UK) and the Netherlands (NL) and identify determinants of positive childbirth experiences in both countries.

**Methods:**

Women who gave birth in the UK (*n* = 1303) or the NL (*n* = 900) between January 2017 and December 2020 who filled in the cross‐sectional Babies Born Better survey were included in this study. Fully adjusted logistic regression models were used to assess differences in the odds of a positive childbirth experience between the two countries. Hierarchical logistic regression analyses were performed to identify determinants of a positive childbirth experience, including socio‐demographic factors, pregnancy and childbirth outcomes, and care‐related determinants.

**Results:**

Respondents giving birth in the UK had decreased odds of a positive childbirth experience compared to NL respondents (66% vs. 85%, AOR 0.45, CI 0.35–0.57). Significant determinants for a positive childbirth experience were multiparity, absence of pregnancy complications, a spontaneous vaginal birth, and giving birth at home. UK respondents who had a planned caesarean section had a higher likelihood of reporting a positive childbirth experience when adjusted for confounders. Having a doctor as the primary birth care provider was less likely to be associated with a positive childbirth experience in the UK.

**Conclusions:**

Most women in both the NL and the UK reported positive childbirth experiences, but NL respondents were more likely to do so. Determinants of a positive birth experience were mostly factors associated with uncomplicated labor and birth, or linked with fulfilled choices and with being multiparous.

## 1 Introduction

The experience of childbirth is an important part of a woman's journey into motherhood [1]. The World Health Organization (WHO) acknowledges that a positive childbirth experience is a critical aspect of ensuring high‐quality maternity care [1]. Moreover, a positive childbirth experience can help promote better physical and emotional health outcomes for both the mother and the baby, and can foster strong bonds between mother and child [2, 3]. For example, women who have a positive childbirth experience are more likely to initiate and continue breastfeeding, which can have long‐term health benefits for both mother and baby [4]. Furthermore, women who have negative childbirth experiences are at higher risk of postpartum depression and other mental health issues [5, 6]. Overall, the childbirth experience can have a ripple effect on many aspects of a woman's life and her baby. As such, understanding the determinants that contribute to a positive childbirth experience is crucial for improving maternity care.

To reach a positive childbirth experience, it is important for many women to have a labor and birth that meets their personal and socio‐cultural expectations [7]. This means giving birth to a healthy baby in a safe environment, both clinically and psychologically, with practical and emotional support from birth companions and competent and kind staff [7]. Furthermore, factors that contribute to a positive childbirth experience include care that is respectful and individualized, with effective communication, access to midwifery continuity of care models, and good integration between services when medical care is needed or wanted [8].

In a previous research article, we hypothesized that women from the Netherlands (NL) and the United Kingdom (UK) who gave birth during the COVID‐19 pandemic might have had a less satisfactory childbirth experience compared to those who gave birth before the pandemic, due to pandemic‐related restrictions such as limited access to birth companions and midwifery continuity of care models, and a potentially less safe psychological environment [9]. To our surprise, the findings showed that women in both countries who gave birth during the pandemic were as likely or more likely to report a positive childbirth experience compared to those who gave birth before COVID‐19 [9]. Furthermore, women giving birth in the NL were much more likely to rate their childbirth experience as positive than women giving birth in the UK. This is different from the results of two surveys about childbirth experiences in the NL and the UK conducted in 2001 in which women in the UK were more positive [10, 11]. Thus, the aim of this study was to further investigate this recently reported difference in childbirth experience between women in the UK and the NL, with a focus on examining potential confounding factors possibly attenuating this association. Furthermore, we aimed to examine determinants that are associated with a positive childbirth experience, and to see whether these differ between the two countries.

## 2 Material and Methods

### 2.1 Study Design

We conducted a cross‐sectional survey in the UK and the NL among women who gave birth between January 2017 and December 2020.

### 2.2 Measurement Tool

We used responses to the international Babies Born Better questionnaire (B3 survey) version 3. The purpose of the B3‐survey was to evaluate and interpret variations in the quality of maternity care at both national and international levels based on women's self‐report. The questionnaire was available in 25 languages.

### 2.3 Respondents and Data Collection

The digital B3‐survey version 3 was made available on the official B3survey website and was promoted by researchers, maternity care organizations, service‐user organizations, hospitals, and midwifery practices through various social media platforms. For this study, eligible participants were women who had given birth in either the NL or the UK during the period of 2017–2020. All the participants were above 18 years old and gave digital informed consent before they started the questionnaire. The questionnaire was completely anonymous; respondents were aware that they could not withdraw their data once they had submitted their responses.

### 2.4 Variables

#### 2.4.1 Main Outcome: Positive Childbirth Experience

The dependent variable, childbirth experience, was initially measured using a 5‐pointscale.

Likert scale ranging from “mostly a very good experience” to “mostly a very bad experience”. This variable was dichotomized into two categories: positive and not positive. Positive contained responses “mostly good” and “quite good” and not positive contained responses “some good, some bad,” “quite bad,” “mostly bad.” This dichotomization of childbirth experience was determined by consensus among the authors and was based on previous research into childbirth experience before and during the COVID‐19 pandemic in NL and the UK [9].

#### 2.4.2 Determinants

The independent, or explanatory, variables were categorized into three groups: (1) socio‐demographic determinants, including parity (primiparous/multiparous), age, migrant status (yes/no), civil status (in a relationship/other), education (secondary/tertiary/university), and self‐reported standard of living (above average/average/below average); (2) pregnancy and childbirth outcomes, including prematurity, pregnancy complications (minor medical problems/minor non‐medical problems/severe medical problems/severe non‐medical problems), and mode of birth (spontaneous vaginal birth/assisted vaginal birth/planned caesarean section/unplanned caesarean section/other); and (3) care‐related determinants, including place of birth (home/birth center/hospital) and the care provider that made most decisions together with the woman during childbirth (doctor/midwife/a combination of care providers/other). Our study focuses on the lived experience of our participants. We did not have access to their medical records, and we explicitly did not ask women what conditions they experienced. We therefore did not examine whether the participants' answers about the variables aligned with particular medical conditions.

### 2.5 Statistical Analysis

Frequencies and percentages were calculated for all categorical variables. We performed univariable logistic regression analyses to examine the associations between explanatory variables and a positive childbirth experience, and calculated odds ratios (OR) with 95% confidence intervals (CI).

Subsequently, we conducted a hierarchical logistic regression analysis using a conceptual hierarchical framework, in line with the approach proposed by Victora et al. [12] To correctly examine determinants of a certain outcome, Victora et al. proposed taking into account the often interrelated hierarchical associations between the determinants by building a multivariable hierarchical model incorporating the assumed framework of the factors which one intends to control for [12]. Therefore, the hierarchical framework involved the sequential entry of determinants based on their assumed direct or indirect influence on all the other factors being studied. The first step included sociodemographic determinants, as we assumed that these factors may directly or indirectly affect all other factors in the model. The second step involved perinatal outcome determinants, and the third and final step incorporated care‐related determinants. Variables which had a statistically significant association (*p* < 0.05) with a positive childbirth experience at each level of the predefined hierarchical model were considered determinants of a positive childbirth experience. At each step, the corresponding variables were simultaneously entered, and the determinants that showed a significant association with a positive childbirth experience were kept for subsequent steps [12]. The reported adjusted odds ratios (aORs) represent the results obtained from the final risk model. We tested the robustness of our hierarchical model by rearranging the sequential entry of the determinant group (i.e., care‐related determinants were now entered in step 2 before entering perinatal outcome determinants in step 3).

Finally, using logistic regression models, we examined whether there was a statistically significant difference between the NL and the UK in the odds of positive childbirth experiences after adjustment for potential confounders. We adjusted for confounders selected using the change‐in‐estimate criterion [13]. That means potential confounders were retained if they altered the effect estimates of the main determinant (country) by more than 5%.

All analyses were based on complete case analysis and conducted using SPSS, version 28.0 for Windows (SPSS Inc., Chicago, IL, USA).

## 3 Results

There were 2203 respondents, of whom 1303 (60%) gave birth in the UK and 900 (40%) in the NL. Socio‐demographics, pregnancy and labor characteristics, and childbirth experiences are shown in Table 1. The median age of women during birth was 31 years in the UK and 32 years in the NL. The respondents in the UK were more often primiparous (56%) than those in the NL (46%). The Dutch sample had a higher rate of spontaneous vaginal births (79%) and homebirths (32%) compared to the UK sample (60%; 14% respectively). The NL had a higher percentage of women experiencing a positive childbirth experience (85%) compared to the UK (66%).

**TABLE 1:** Socio‐demographic, pregnancy, labor characteristics, and birth experience of the respondents who completed the Babies Born Better survey in 2017–2020 (*n* = 2203).

Characteristics		UK	Netherlands
		*n* (%)	*n* (%)
Age	≤ 25 years	154 (11.8)	63 (7)
	26–30 years	450 (34.5)	301 (33.4)
	31–35 years	475 (36.5)	384 (42.7)
	≥ 36 years	224 (17.2)	152 (16.9)
Parity	Primiparous	725 (55.6)	417 (46.3)
	Multiparous	578 (44.4)	483 (53.7)
Migrant	Yes	150 (11.5)	51 (5.7)
	No	1151 (88.5)	848 (94.3)
Civil status	In a relationship	1263 (97.2)	877 (97.6)
	Other	37 (2.8)	22 (2.4)
Standard of life	Above average	731 (56.1)	523 (58.2)
	Average	553 (42.5)	360 (40)
	Below average	18 (1.4)	16 (1.8)
Education	Secondary education or less	151 (11.6)	15 (1.7)
	Tertiary/professional/technical	136 (10.5)	186 (20.6)
	University or equivalent	1016 (78.1)	699 (77.7)
Preterm birth	Yes	75 (5.8)	38 (4.2)
	No	1228 (94.2)	862 (95.8)
Pregnancy complications	Yes	573 (44)	361 (40.1)
	No	730 (56)	539 (59.9)
Giving birth during the COVID‐19 pandemic	Yes	468 (36)	210 (23.4)
	No	833 (64)	687 (76.6)
Type and severity of pregnancy complications	Minor medical problems	381 (65)	224 (60.4)
	Minor non‐medical problems	118 (20.1)	89 (24)
	Severe medical problems	69 (11.8)	51 (13.7)
	Severe non‐medical problems	18 (3.1)	7 (1.9)
Mode of birth	Spontaneous vaginal birth	783 (60.1)	706 (78.5)
	Assisted vaginal birth	209 (16.1)	74 (8.2)
	Planned caesarean section	117 (9.0)	44 (4.9)
	Unplanned caesarean section	174 (13.4)	62 (6.9)
	Other/not clearly indicated	19 (1.5)	13 (1.4)
Place of birth	Home	184 (14.1)	287 (31.9)
	Hospital	887 (68.2)	492 (54.7)
	Birth center	230 (17.7)	121 (13.4)
Care provider during birth	Doctor	165 (12.7)	158 (17.7)
	Midwife	671 (51.7)	518 (58.0)
	A combination of care providers	421 (32.5)	175 (19.6)
	Other	40 (3.1)	42 (4.7)
Birth experience	Mostly a very good experience	624 (48.0)	581 (64.6)
	Mostly quite a good experience	231 (17.8)	181 (20.1)
	Some of it was good and some of it was bad	264 (20.3)	85 (9.5)
	Mostly quite a bad experience	105 (8.1)	31 (3.4)
	Mostly a very bad experience	76 (5.8)	21 (2.3)
Positive birth experience	Yes	855 (65.8)	762 (84.8)
	No	445 (34.2)	137 (15.2)

*Note:* Data were missing in the following variables: migrant status *n* = 3, multiple birth *n* = 4, civil status *n* = 4, standard of life = 2, education level = 2, type of birth = 2, place of birth = 2, health care provider during birth = 13, birth experience = 4.

### 3.1 Determinants Associated With Childbirth Experience

In both the UK and the NL samples, women who had a positive childbirth experience were more often multiparous, university educated, experienced no pregnancy complications, had a spontaneous vaginal birth, gave birth at home, and/or had a midwife as care provider (Table 2). Additionally, in the UK sample, women who self‐reported an above‐average standard of life, delivered at full term, and/or had a planned caesarean section birth were more likely to have a positive childbirth experience. In the NL sample, women who were in a relationship were more likely to have a positive birth experience; although only 24 women (2.6% of the NL sample) reported that they were not in a relationship.

**TABLE 2:** Association between different variables and a positive childbirth experience within the United Kingdom and within the Netherlands.

Variables		United Kingdom				Netherlands			
		Negative birth experience, *n* (%)	Positive birth experience, *n* (%)	Crude odds ratio^a^ [95% CI]	Adjusted odds ratio in final model^b^ [95% CI]	Negative birth experience, *n* (%)	Positive birth experience, *n* (%)	Crude odds ratio^a^ [95% CI]	Adjusted odds ratio in final model^b^ [95% CI]
Sociodemographic factors									
Parity	Nulliparous	310 (43.8)	397 (56.2)	0.35 (0.27–0.45)**	0.61 (0.45–0.83)**	87 (21.3)	322 (78.7)	0.37 (0.25–0.55)***	0.62 (0.39–0.96)*
	Multiparous	120 (21.4)	441 (78.6)	Ref	Ref	42 (9.0)	424 (91.0)	Ref	Ref
Age	≤ 25 years	58 (39.2)	90 (60.8)	0.82 (0.56–1.20)	NA	9 (14.5)	53 (85.5)	1.06 (0.49–2.31)	NA
	26–30 years	152 (34.5)	289 (65.5)	Ref		45 (15.3)	249 (84.7)	Ref	
	31–35 years	159 (34.3)	304 (65.7)	1.01 (0.76–1.32)		52 (14)	319 (86)	1.11 (0.72–1.71)	
	≥ 36 years	61 (28.2)	155 (71.8)	1.34 (0.94–1.91)		23 (15.5)	125 (84.5)	0.98 (0.57–1.70)	
Migrant	No	382 (33.9)	744 (66.1)	Ref	NA	118 (14.3)	707 (85.7)	Ref	NA
	Yes	48 (33.8)	94 (66.2)	1.01 (0.70–1.45)		11 (22)	39 (78)	0.59 (0.30–1.19)	
Civil status	In a relationship	415 (33.6)	819 (66.4)	Ref	NA	122 (14.3)	732 (85.7)	Ref	NA
	Other	15 (44.1)	19 (55.9)	0.64 (0.32–1.28)		7 (33.3)	14 (66.7)	0.34 (0.13–0.84)*	
Standard of life	Above average	234 (32.9)	477 (67.1)	Ref	Ref	66 (13)	442 (87)	Ref	NA
	Average	185 (34.3)	355 (65.7)	0.94 (0.74–1.19)	0.85 (0.64–1.12)	59 (16.8)	292 (83.2)	0.74 (0.51–1.08)	
	Below average	11 (64.7)	6 (35.3)	0.27 (0.10–0.73)*	0.23 (0.07–0.77)*	4 (25)	12 (75)	0.45 (0.14–1.43)	
Education	University or equivalent	316 (31.9)	675 (68.1)	Ref	NA	88 (13)	590 (87)	Ref	NA
	Tertiary/professional/technical	54 (40.3)	80 (59.7)	0.69 (0.48–1.00)		39 (21.4)	143 (87.6)	0.55 (0.36–0.83)**	
	Secondary education or less	60 (42)	83 (58)	0.65 (0.45–0.93)*		2 (13.3)	13 (86.7)	0.97 (0.22–4.37)	
Perinatal outcomes									
Preterm birth	No	393 (32.8)	805 (67.2)	Ref	NA	121 (14.4)	718 (85.6)	Ref	NA
	Yes	37 (52.9)	33 (47.1)	0.44 (0.27–0.71)***		8 (22.2)	28 (77.8)	0.59 (0.26–1.33)	
Pregnancy complications	No	201 (28.2)	513 (71.8)	Ref	Ref	60 (11.4)	468 (88.6)	Ref	Ref
	Yes	229 (41.3)	325 (58.7)	0.56 (0.44–0.70)***	0.58 (0.44–0.77)***	69 (19.9)	278 (80.1)	0.52 (0.35–0.75)***	0.79 (0.52–1.19)
Mode of birth	Spontaneous vaginal birth	148 (19.1)	625 (80.9)	Ref	Ref	66 (9.5)	632 (90.5)	Ref	Ref
	Assisted vaginal birth	128 (62.1)	78 (37.9)	0.14 (0.10–0.20)***	0.31 (0.21–0.45)***	27 (37.5)	45 (62.5)	0.17 (0.10–0.30)***	0.38 (0.21–0.70)**
	Planned caesarean section	30 (25.9)	86 (74.1)	0.68 (0.43–1.07)	1.87 (1.06–3.29)*	8 (18.6)	35 (81.4)	0.46 (0.20–1.03)	0.53 (0.20–1.39)
	Unplanned caesarean section	124 (71.7)	49 (28.3)	0.09 (0.06–0.14)***	0.23 (0.15–0.35)***	28 (45.2)	34 (54.8)	0.13 (0.07–0.22)***	0.25 (0.13–0.48)***
Care‐related factors									
Place of birth	Home	4 (2.2)	176 (97.8)	Ref	Ref	7 (2.5)	277 (97.5)	Ref	Ref
	Birth center	43 (19)	183 (81)	0.10 (0.03–0.28)***	0.13 (0.04–0.37)***	8 (6.7)	112 (93.3)	0.35 (0.13–1.00)	0.45 (0.16–1.23)
	Hospital	383 (44.4)	479 (55.6)	0.03 (0.01–0.08)***	0.07 (0.02–0.19)***	114 (24.2)	357 (75.8)	0.08 (0.04–0.17)***	0.14 (0.06–0.33)***
Care provider during birth	Midwife	136 (20.5)	528 (79.5)	Ref	Ref	43 (8.4)	470 (91.6)	Ref	Ref
	Doctor	84 (53.5)	73 (46.5)	0.22 (0.16–0.32)***	0.38 (0.23–0.63)***	31 (21.1)	116 (78.9)	0.34 (0.21–0.57)***	1.46 (0.76–2.83)
	A combination of care providers	202 (49.5)	206 (50.5)	0.26 (0.20–0.34)***	0.58 (0.42–0.81)**	50 (28.7)	124 (71.3)	0.23 (0.15–0.36)***	0.78 (0.46–1.33)
	Other	8 (20.5)	31 (79.5)	1.00 (0.45–2.22)	0.43 (0.16–1.19)	5 (12.2)	36 (78.8)	0.66 (0.25–1.77)	1.09 (0.35–3.39)

*Note:* Significance of bold value indicates statistical significance of * when *p* < 0.05, ** when *p* < 0.01 and ** when *p* < 0.001.

Abbreviation: NA, not applicable, the respective predictor variable was not included in the final model.

^a^ Univariable analyses.

^b^ Adjusted for all variables included in the final model.

Table 2 also presents the outcomes of the final multivariable hierarchical regression model, identifying the independent determinants of a positive childbirth experience. The sociodemographic factors that remained significant were parity (in both the UK and NL samples) and standard of life (UK sample). In both the UK and the NL, nulliparous women had decreased odds of having a positive childbirth experience (UK AOR 0.61, 95% CI 0.45–0.83; NL AOR 0.62, CI 0.39–0.96). Among UK women, a below‐average standard of life was independently associated with a negative childbirth experience (AOR 0.23, CI 0.07–0.77).

Women in the UK with pregnancy problems had decreased odds of having a positive childbirth experience (AOR 0.58, CI 0.44–0.77); whereas this association was not statistically significant in the NL (AOR 0.79, CI 0.52–1.19). Both in the UK and the NL, women who had an assisted vaginal birth (UK AOR 0.31, CI 0.21–0.45; NL AOR 0.38, CI 0.21–0.70) or an unplanned caesarean section (UK AOR 0.23, CI 0.15–0.35; NL AOR 0.25, CI 0.13–0.48) had decreased odds of experiencing a positive childbirth experience compared to women with a spontaneous vaginal birth. Conversely, in the UK, women who had a planned caesarean section were more likely to have a positive childbirth experience compared to those with a spontaneous vaginal birth (AOR 1.87, CI 1.06–3.29); whereas this relationship was not observed in the NL (AOR 0.53, CI 0.20–1.39).

In comparison to a home birth, a hospital birth was associated with a 93% lowered odds of a positive childbirth experience in the UK and an 86% lowered odds in the Netherlands (UK AOR 0.07, CI 0.02–0.19; NL AOR 0.14, CI 0.06–0.33) indicating strong effect sizes. Furthermore, we observed the same pattern of results for a birth center birth in the UK when compared to a home birth (AOR 0.13, CI 0.04–0.37). In the UK, women who had a doctor as their care provider had decreased odds of experiencing a positive childbirth experience (AOR 0.38, CI 0.23–0.63), whereas in the NL, this was not the case (AOR 1.46, CI 0.76–2.83). In both countries, a combination of care providers was associated with decreased odds of positive childbirth experience in comparison with a midwife as care provider, although not significant in the NL (UK AOR 0.26, CI 0.42–0.81; NL AOR 0.78, CI 0.46–1.33).

Re‐arranging the order of the sequential entry of the determinants did not alter the results.

### 3.2 The Effect of Country on a Positive Childbirth Experience

Respondents who gave birth in the UK had decreased odds of having a positive childbirth experience (OR 0.34, CI 0.27–0.43) compared to women who gave birth in the NL (Table 3). The confounders selected by the change‐in‐estimate criterion were place of birth and mode of birth. The effect remained statistically significant even after controlling for these confounders (AOR 0.45, CI 0.35–0.57).

**TABLE 3:** The association of country with a positive versus negative childbirth experience.

Variables		Negative birth experience, *n* (%)	Positive birth experience, *n* (%)	Crude odds ratio [95% CI]	*p*	Adjusted odds ratio^a^ [95% CI]	*p*
Country	Netherlands	137 (23.5)	762 (47.1)	Ref		Ref	
	United Kingdom	445 (76.5)	855 (52.9)	**0.34 (0.27–0.43)**	**< 0.001**	**0.45 (0.35–0.57)**	**< 0.001**

^a^ Adjusted for confounders selected by the change‐in‐estimate procedure [13]. Selected confounders included place of birth and mode of birth.

## 4 Discussion

### 4.1 Main Findings

In the current study, most women reported a positive childbirth experience, although a significant minority did not. Respondents from the NL had a significantly better perception of their childbirth experience than those in the UK, even after controlling for confounders. The key determinants for a positive childbirth experience in both countries were multiparity, having a spontaneous vaginal birth, and giving birth at home. In the UK, women who had a planned caesarean section were more likely to report a positive childbirth experience, although having a doctor as the care provider was associated with the opposite. Furthermore, for women in the UK, pregnancy problems and below average standard of life were associated with decreased odds of having a positive childbirth experience. These factors were not significant in the NL.

### 4.2 Strengths and Limitations

This is the first survey comparing UK and NL women's childbirth experiences since 2001. The B3 survey was available in 25 languages, but women who were unable to read their language or who lacked access to smartphones or computers may have been excluded. It is a self‐report instrument, so it may be prone to recall bias. However, respondents completed the questionnaire within 3 years of birth, and there is good evidence that maternal recall tends to be accurate [14]. Due to applying convenience sampling, certain socio‐economic groups and obstetric and perinatal outcomes had low prevalence rates, reducing the generalizability of this study to broader populations in the UK and the NL. Because national data was not available or reliable enough, weighting of the sample data was not possible. This was partly because the Dutch perinatal database experienced data submission issues from primary midwifery practices, leading to an underrepresentation of data from primary care.

We assessed having a positive childbirth experience with one question in the survey that is designed to be a simple “common sense” measure, rather than a series of psychometric experience items. This is because the questionnaire was deliberately made short so that many women would be likely to complete it when recruited via social media. Furthermore, the responses of the women to the survey questions are based on their experiences, which may differ from the objective reality in certain aspects. For instance, a woman may indicate that she shared decision making with a midwife during labor, because this is who she saw, when, in fact, the midwife could have been consulting with a doctor for some of the options that were made available. Our results therefore do not reflect midwife‐led care or obstetrician‐led care as defined in other scientific literature.

The choice for dichotomization was based on consensus among the authors. It is in line with the earlier published article about childbirth experiences before and during the COVID‐19 pandemic, but it is not the same measure as for previous childbirth experience surveys in the NL and the UK in 2001 [9, 10, 11].

### 4.3 Interpretation

In comparison to the earlier survey in 2001 rates of positive birth experiences in the UK remained relatively stable between 2001 and 2020, while women in the Netherlands reported more positive childbirth experiences than in 2001 [10, 11]. Moreover, women had a more negative childbirth experience in the UK than in the Netherlands between 2017 and 2020. One possible explanation for this finding could be the improvements in the Dutch maternity care system in the last three decades. For example, since 2001 midwives and obstetricians have formed Maternity Care Collaborations, it has become easier for women to receive pain relief during labor if desired, and shared‐decision making has been introduced [15, 16, 17]. In contrast, the maternity care system in the UK is increasingly under pressure due to severe staff shortages leading to reports of increased burnout and moral distress among midwives, of a lack of freedom of choice for women, of an increase in adverse outcomes, and of women and families who are traumatized and ignored [18]. Obstetricians and midwives in the UK work in integrated maternity care organizations (NHS Trusts). There is an ongoing effort in the Netherlands to integrate primary midwife‐led care and secondary obstetrician‐led care to enhance continuity of care, safety and women's experiences [19]. However, our results suggest that a more integrated maternity care system such as in the UK is no guarantee for improved client experiences, even though good collaboration within and between maternity care organizations is important for good quality maternity care [20].

Factors that are associated with a more straightforward pregnancy and birth tended to be associated with positive experiences, with the one exception being women who had a planned caesarean section in the UK. This is noteworthy, since receiving care by a doctor alone decreased the odds of a positive experience, and out of hospital birth enhanced the odds. The low numbers of women undergoing planned caesareans in this study may have contributed to this finding. However, women having unexpectedly complex vaginal births may associate the switch from midwife to doctor involvement with fear, disappointment, or even trauma [21]. Other studies have noted that the element of control involved in choosing both a home birth and a planned caesarean section is associated with better outcomes [22, 23, 24]. It is likely that women who do not want or need a caesarean section are more likely to have a better experience in a birth center or at home, with midwives, while those choosing planned caesarean section are more likely to feel in control and therefore have a positive experience as well [25]. In contrast, women who prefer physiological birth may not have the experience they planned in hospital and with doctors, and this may be influencing the findings. This implies a need for maternity care providers to be skilled, confident, and empowered to support women who prefer a physiological labor and birth to have the best chance of doing so safely (including offering out of hospital options), as well as taking the informed choice of women for planned caesarean section seriously.

Prior studies have linked multiparity with improved childbirth experiences [10, 26]. Although multiparous women have a higher chance of a straightforward uncomplicated birth, our corrections for assisted vaginal or caesarean birth did not change the “multiparity benefit.” [27] However, we did not have data on labor induction and augmentation, or episiotomy, for instance, and these factors could have changed this finding. There may be some psychological factors as well, since multiparous women know what to expect, and may feel more empowered to assert their preferences and make informed decisions [26, 28].

Finally, we found that respondents who answered that a midwife made most decisions during birth with them were more satisfied with their birth experience, even after controlling for confounders, in line with other studies [29]. However, in contrast to other literature, the effect disappeared for Dutch women in multivariate analysis. This suggests that, while women who give birth in a hospital or have pregnancy problems in the NL are more likely to have a doctor as their healthcare provider, in NL the doctor alone does not independently contribute to an increased odds of a negative childbirth experience. In contrast, in the UK, a doctor as care provider remained independently associated with a negative birth experience in the multivariable analysis, despite the positive impact of planned caesarean section, which is always associated with obstetrician‐led care. These differences between and within the NL and the UK are items for future research, for example by looking at the qualitative responses to the B3 survey and by studying how women interpret the survey questions and what they consider midwifery of obstetrician led care. In both countries, women who were attended by multiple healthcare providers had increased odds of a negative childbirth experience, which is supported by other literature [25]. If more care providers are involved a birth is more often not straightforward. Women can find transfers between care providers during childbirth stressful, and disruptive to continuity of care, which may influence their experience [30]. Changes in care provider may also influence the kind of childbirth choices that can be made, including place and mode of birth. Additionally, communication between maternity care providers during a transfer of care can be a concern [31].

### 4.4 Implications for Practice

This study demonstrates which factors influence the odds of having a positive childbirth experience. Acknowledging the complex experience of women regarding their childbirth, translating these factors into ready‐made implications for practice is challenging. However, many of the identified determinants are linked to respectful, compassionate and individualized care. This should be prioritized for every woman and birthing person throughout labor and birth, regardless of the characteristics a particular woman may have that could increase or decrease her chances of a positive childbirth experience [1].

## 5 Conclusion

In our survey most respondents had a positive childbirth experience, though a significant minority did not. Respondents from the NL had a significantly better perception of their childbirth experience than those from the UK. Determinants of a positive birth experience were mostly factors associated with uncomplicated labor and birth, or linked with fulfilled choices and with being multiparous. For improving childbirth experience, irrespective of the country or specific determinants involved, maternity care should prioritize providing respectful, compassionate, individualized care to every woman and birthing person, throughout labor and birth.

## Ethics Statement

In the NL the study was submitted to the Medical Ethics Review Committee of the VU University Medical Centre (reference number 2020.255). No ethical approval was needed, since the Medical Research Involving Human Subjects Act did not apply to this study, as there was considered to be no infringement on the physical and/or psychological integrity of the participants. In the UK the study was approved by the University of Central Lancashire (UCLan) Committee for Ethics and Integrity (STEMH 449 Amendment_1Jun20).

## Conflicts of Interest

The authors declare no conflicts of interest.

## Data Availability

All data underlying the results (B3 dataset) are available via the UK Data Services (UKDS) ReShare repository. DOI: 10.5255/UKDA‐SN‐855862 Link: https://reshare.ukdataservice.ac.uk/855862/. Access: Safeguarded access—permission only.
